# Specific Aspects of Eye Movement Reactions as Markers of Cognitive Control Disorders in Patients with Obsessive-Compulsive Disorder (Review)

**DOI:** 10.17691/stm2022.14.2.08

**Published:** 2022-03-28

**Authors:** G.M. Khayrullina, V.V. Moiseeva, O.V. Martynova

**Affiliations:** Junior Researcher; Institute of Higher Nervous Activity and Neurophysiology, Russian Academy of Sciences, 5A Butlerova St., Moscow, 117485, Russia; PhD Student, Center for Neuroeconomics and Cognitive Research, Institute of Cognitive Neurosciences; National Research University Higher School of Economics, 20 Myasnitskaya St., Moscow, 101000, Russia; Senior Researcher, Deputy Director, Center for Neuroeconomics and Cognitive Research, Institute of Cognitive Neurosciences; National Research University Higher School of Economics, 20 Myasnitskaya St., Moscow, 101000, Russia; Head of the Laboratory of Human Higher Nervous Activity; Institute of Higher Nervous Activity and Neurophysiology, Russian Academy of Sciences, 5A Butlerova St., Moscow, 117485, Russia; Senior Researcher, Center for Neuroeconomics and Cognitive Research, Institute of Cognitive Neurosciences; National Research University Higher School of Economics, 20 Myasnitskaya St., Moscow, 101000, Russia

**Keywords:** obsessive-compulsive disorder, cognitive control, selective attention, inhibitory control, working memory, eye movements, oculography, eye tracking

## Abstract

Multiple studies in patients with obsessive-compulsive disorder (OCD) became the basis for revealing selective attention, inhibitory control, and working memory impairments, which correlates with an imbalance in the activity of the cortico-striatal-thalamic-cortical circuit associated with maintenance of cognitive control functions. Patients with OCD often demonstrate changes in the parameters of target-oriented eye movement reactions being a consequence of a possible impairment of the cognitive control neurophysiological framework. This review summarizes and analyzes data on cognitive control disorders in OCD obtained with eye movement recording techniques.

It was established that the most often used are smooth pursuit eye movements tasks, memory-guided saccades, and anti-saccadic tasks. Data on smooth pursuit eye movements tasks and memory-guided saccades are contradictory, although they partially confirm selective attention and working memory impairment. Most studies on the anti-saccadic task identified impaired inhibitory control in patients with OCD. Similar disorders in form of increased latency and higher error rate in anti-saccades were also noted in the patients’ first-degree relatives, which allows considering such disorders as manifestations of the endophenotype associated with the underlying risk of OCD. Future confirmation of these results in experiments using complex anti-saccadic tasks with images of various modalities (taking into account the increased anxiety in patients with OCD as the disorder basis) might contribute to validation of the OCD-specific markers.

## Introduction

Most people have intrusive thoughts or overwhelming behavioral urges. When such states become frequent, excessive, and life-disruptive, they are diagnosed as the obsessive-compulsive disorder (OCD). OCD is highly prevalent in the general population (1–3%) and is associated with significant comorbidity and heterogeneity in clinical manifestations [1, 2].

The World Health Organization called OCD one of the top ten incapacitating disorders. Patients with OCD try to avoid situations that make them feel uncomfortable, even with no real threat. This can lead to a decrease in social interactions and quality of life, as well as affect social and economic development of society in general. Most people with OCD do not seek treatment for years due to stigmatization, despite they understand meaninglessness of intrusive thoughts and/or ineffectiveness of rituals [3, 4].

Obsessive-compulsive disorder can be considered a “model” disorder to study rigidity of mental processes in both neurotypical individuals and individuals with other mental diseases. Although knowledge about OCD developed, some questions on the triggering mechanisms thereof remain unclear.

In studies, where neuroimaging, neuropsychological, and pharmacological techniques were applied, investigators demonstrated that OCD is associated with a dysfunction of the cortico-striatal-thalamic-cortical circuit, based on an imbalance between excitatory glutamatergic and inhibitory GABA-ergic systems, as well as on an imbalance of serotonin and dopamine, which is accompanied by impaired cognitive and emotional control [5, 6].

**Figure 1.**
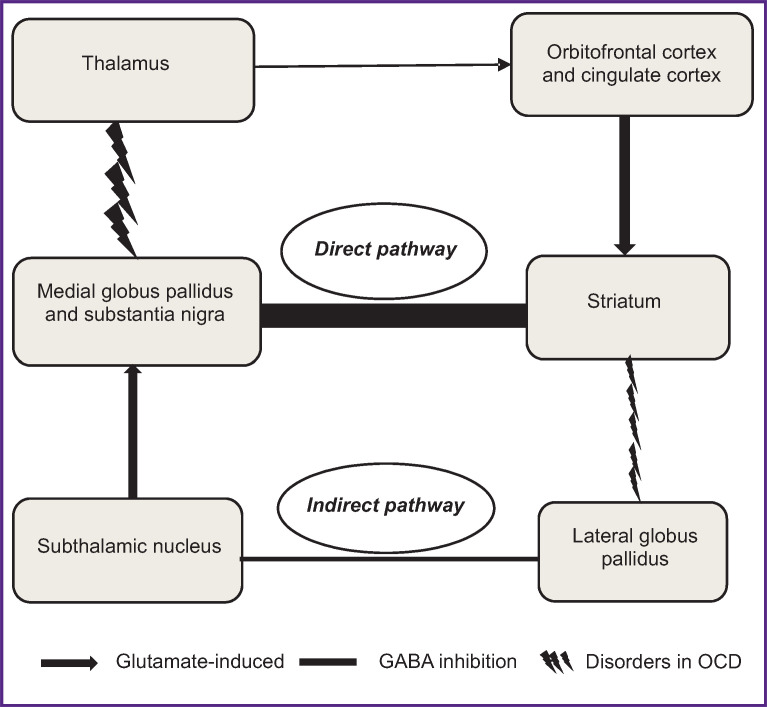
Cortico-striatal-thalamic-cortical circuit in obsessive-compulsive disorder (adapted from [7])

The study of neurotransmitters in patients with OCD revealed an imbalance between direct and indirect pathways in the cortico-striatal-thalamic-cortical circuit [7] (Figure 1). It was suggested that the balance shift towards the direct pathway associated with an increased glutamate concentration might lead to development of OCD symptoms [8]. Thus, hyperstimulation of the orbitofrontal cortex and the anterior cingulate cortex mediates exaggerated feeling of danger, leading to a constant attention to the perceived threat, which is followed by development of rituals to eliminate the said threat. Alternatively, striatal dysfunction is assumed to reduce inhibition of the lateral globus pallidus, resulting in the increased inhibition of the subthalamic nucleus, effectively reducing excitation of the medial globus pallidus and substantia nigra, and by so doing it increases excitation from the thalamus to the orbitofrontal cortex and anterior cingulate cortex [9] (see Figure 1). Predominant activity of the direct pathways centers compared with the indirect pathways centers leads to cognitive control functions impairment, which might be the basis for the OCD symptoms [10-12].

One of the largest reviews [13] summarizes the results of over 900 OCD studies using positron emission tomography as well as structural and functional magnetic resonance imaging (fMRI). The hypothesis is that in case of OCD initial dysfunction of the caudate nucleus leads to ineffective sensory gating at the thalamus level, followed by hyperactivity of the orbitofrontal cortex (in obsessions) and the anterior cingulate cortex (in compulsions). Here, the authors emphasize that the mentioned changes in the activity of the brain structures primarily correlate with impairments of processing visual modality spatial stimuli and control of eye movement reactions in patients with OCD [13]. A more recent review [3] also discusses dysfunction of the cortico-striatal circuit. Most of the studies analyzed by the authors established excessive activation of the orbitofrontal cortex and basal ganglia in case of symptom provocation and further reduction thereof after patients’ treatment with selective serotonin reuptake inhibitors and cognitive-and-behavioral psychotherapy. Finally, in the review [14] the following was stated for patients with OCD, in addition to disruption of the cortico-striatal-thalamic-cortical circuit: structural changes and altered functional activity of the limbic areas — the amygdala, the parietal and occipital cortex of the brain, and the cerebellum.

Thus, most neurophysiological studies highlight impairment of the activity and functional relationship of the basal ganglia, thalamus, and medial prefrontal cortex in patients with OCD [15, 16]. Neuropsychological studies confirm worsening of the situation with the cognitive control: working memory (WM), attention, inhibitory control, verbal flexibility, planning, and decision-making. As there are data on impairment of the eye movement control [17, 18] in OCD, the technique of recording eye movements is recognized as a non-invasive neurophysiological approach to studying cognitive impairment in these patients [19]. Non-invasive registration of eye movements is achievable with the use of oculographic electrodes — oculography, as well as an infrared camera that records eye movements by tracking the pupil — the eye tracking technique [20]. Compared to fMRI, these techniques are less resource-consuming and provide for a direct (rather than indirect as in fMRI) evaluation of the neurophysiological features of cognitive control by successful performance of tasks on saccades or smooth pursuit eye movements [21, 22].

**The aim of this review** is to summarize and analyze the accumulated data on cognitive control disorders in OCD, which were received using oculography and eye tracking.

**Figure 2.**
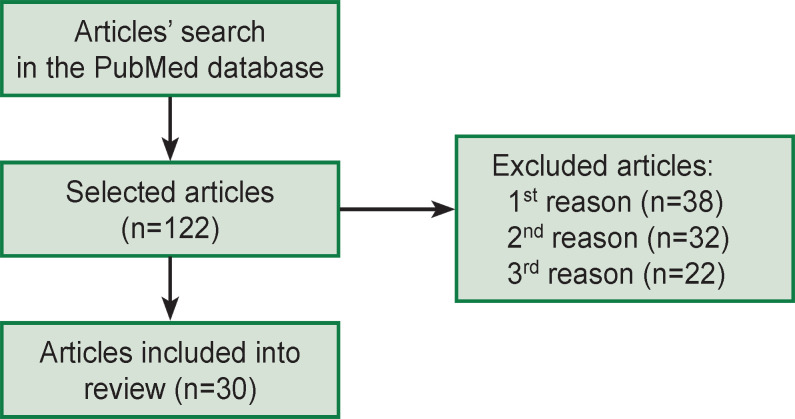
Diagram demonstrating stages of articles’ search and selection in accordance with the PRISMA recommendations

Articles were searched in the PubMed database (https://pubmed.ncbi.nlm.nih.gov/). Key words for the search included “obsessive-compulsive disorder” AND “eye movements” OR “eye tracker” OR “saccade” OR “oculography”. There were 122 articles found, 30 of them were selected as they provided data on specific aspects of eye movement reactions in OCD (Figure 2). Articles exclusion criteria: 1^st^ reason is associated with studying of other patients (OCD was mentioned only in the discussion); 2^nd^ reason — there was no eye tracking and/or oculography data; 3^rd^ reason — data were collected for a specific population (without OCD diagnosis, but with particular signs of the disorder). Among 30 selected studies, only one review [23] and one meta-analysis, combined with experimental data of the authors, are exclusively devoted to one type of eye movement tasks [24]. A search for articles using techniques for eye movements recording in patients with OCD in databases in Russian had no effect. A summary of studies on cognitive control of eye movements in case of OCD is provided in Appendix 1.

The review [23], published in 2011, most widely covered the data on eye movements in case of OCD accumulated by that time. The authors concluded that patients with OCD demonstrated only rather non-specific impairments in performing simple tasks (minor impairments in smooth pursuit eye movements and increased latency in performing anti-saccade tasks) compared to patients with schizophrenia. Thus, there is a need to develop complex tasks to identify specific disorders in case of OCD, for their subsequent use as reliable tools for diagnostic testing and classification of various types of this disease. The authors could not find more recent reviews, so this article is an attempt to fill this gap.

## Obsessive-compulsive disorder

Obsessions and/or compulsions are an essential feature of OCD. Obsessions are haunting, involuntary, and most often stereotypic ideas, images, or urges accompanied by anxiety in almost all the cases. A patient with OCD unsuccessfully tries to resist them, but such attempts are in vain. Compulsions are characterized by repetitive stereotypic behavior and/or mental acts. A patient with OCD performs those acts in response to

an obsessive thought to reach “comfort”. Such actions neither are performed for the sake of enjoyment nor are attributable to useful tasks. They serve as a means to prevent an unpleasant event which, as the patient thinks, might otherwise occur harming him or he might harm other persons. Anxiety is almost always attributable to OCD; anxiety becomes more expressed if compulsive actions are suppressed [25].

In studies based on the factorial and analytical approach [26, 27], a four-factor model of OCD symptoms was consistently developed, and it includes the following:

concerns about contamination accompanied by washing or cleaning;

fears of harming oneself or other persons accompanied by checking;

obsessive aggressive or sexual thoughts accompanied by mental rituals;

anxiety related to breaking symmetry accompanied by pattern development or counting.

Historically, OCD was classified as an anxiety disorder. In the ICD-10 and DSM-4 classifications, it was categorized as “Neurotic, stress-related, and somatoform disorders”, as compulsive behavior represents an attempt to relieve anxiety caused by obsessions [15]. However, from neurobiological and clinical point of view, OCD differs from typical anxiety disorders in several ways, which was the basis for its exclusion from this category [28-30]. The DSM-5 and ICD-11 classifications have OCD placed into a separate new category “Obsessive-compulsive and related disorders”, which includes disorders characterized by specific types of experience or repetitive behavior. In addition to OCD, DSM-5 includes body dysmorphic disorder, hoarding disorder, hair-pulling disorder, excoriation disorder, secondary causes of OCD (after-effects of psychoactive substances, drugs, etc.) in this category [31]. In ICD-11, in addition to OCD, this group includes dysmorphic disorder, hoarding disorder, hair-pulling disorder, excoriation disorder, as well as olfactory reference syndrome disorder and hypochondriasis [32].

Similar to OCD, obsessive-compulsive related disorders (OCRDs) are also wide-spread. However, they are not recognizable enough. Such disorders are characterized by repetitive and undesired thoughts or behaviors. Some OCRDs include obsessive thoughts and compulsive behaviors (for instance, body dysmorphic disorder), others have predominantly movement or behavioral symptoms (for example, hair-pulling disorder) [16]. Despite OCDs and OCRDs being similar [33, 34], they have key differences in their neurobiology, diagnosis, and treatment [35, 36].

It is important to differentiate the OCD symptoms, which can be found in some neurological conditions, and veritable OCD. In Huntington’s disease, which is characterized by cognitive degradation and progressive movement impairment, one can identify OCD symptoms which tend to become more expressed as the disease progresses [37]. OCD is one of the most wide-spread psychiatric complications after stroke and traumatic brain injury [38]. Patients with Parkinson’s disease might demonstrate specific behavior called ‘punding’, which is complex, long-term, purposeless, stereotypic behavior that can be misidentified as an OCD sign [39]. In Sydenham’s chorea, it has been noted that practically all patients develop OCD symptoms at an early stage of the disease [40] due to an autoimmune response to streptococcal infection leading to inflammation in the basal ganglia — brain regions involved in the pathogenesis of OCD [41]. The symptoms of Tourette’s syndrome might resemble OCD rituals, especially habit-like compulsions such as touching, tapping, rubbing, and repetition of regular activities [42]. However, it is important to understand that such manifestations in neurological diseases are not caused by clear obsessive thoughts (obsessions).

Based on studies of OCD neuroendophenotypes, a wide range of impairments of cognitive control functions was identified; such impairments result in problems with inhibitory control, selective attention, cognitive flexibility, planning, WM, and error tracking [43]. In present, it is not clear whether these disorders are specific to OCD or can be seen in many neurotic spectrum psychiatric disorders. Moreover, taking into account the fact that OCD is a heterogeneous disease, the appropriateness of using general pathophysiological models of OCD to interpret manifestations of various symptoms of the disease is under question as they might differ in terms of genetics, reactivity to medical treatment, various patterns of manifestation of functional brain activity [44, 45]. There are relatively few OCD studies with these parameters examined as of now.

## Impaired cognitive control functions in patients with obsessive-compulsive disorder demonstrated by eye movement records

Lack of eye movement control in OCD is most often associated with impairment of the cortico-striatal-thalamic-cortical circuit [46-48]. Eye movement reactions are studied within various paradigms: smooth pursuit eye movement (SPEM); memory-guided saccade; anti-saccade. Tasks for visually guided/involuntary saccades and voluntary saccades/prosaccade are used in a complex analysis of eye movement reactions together with an anti-saccade task (Appendix 2). The mentioned paradigms are designed to assess basic eye movement behavior and to measure cognitive control functions such as reaction inhibition, planning, and working memory.

The results of studies devoted to specific aspects of eye movement reactions in OCD are contradictory. In a number of studies, patients with OCD demonstrated differences in eye movement reactions compared to the control group; the differences were represented by an extended latency of saccades and/or an increase in the frequency of errors during an anti-saccade task, as well as a decrease in the SPEM accuracy [49-53]. According to other authors, patients with OCD did not differ from the control group in the frequency of errors when performing anti-saccades and did not have differences in SPEMs [24, 54, 55]. This might be due to a wide variety of paradigms, tasks and goals, which prevents determining the specific nature of the deficiency in eye movement control for this disorder (e.g., see meta-analysis [24]).

It should be noted that the correct interpretation of the research data can provide for distinguishing a number of differential diagnoses due to the fact that the neural centers involved in the control of saccadic and SPEMs differ in some way in terms of their location and function. Areas involved in saccade control include the frontal eye fields, supplementary eye fields, posterior parietal cortex, caudate nucleus, and substantia nigra; SPEMs are controlled by the frontal eye fields, as well as the medial temporal cortex and the medial superior temporal cortex [48].

### Study of smooth pursuit eye movements

SPEMs are smooth eye movements while tracking a small object moving at a constant speed. To perform the task correctly, the eye movement speed must constantly adapt to the speed of the object. The most commonly measured variable while studying SPEMs is the acceleration coefficient (gain), calculated as the ratio of the eye movement speed to the target speed. A decrease in this coefficient might indicate difficulty in matching the eye position and the target speed. Other examined parameters include the spatial difference between the position of the target and the position of the eye and the number of corrective saccades. If the eyes do not reproduce the target movement, the saccade system must compensate for this error using corrective saccades [56].

Neurophysiological mechanisms of the visual target tracking system intersect with the mechanisms of the saccadic movement system. They work integratively [57, 58], despite the fact that some areas involved in the generation of SPEMs differ from the structures that exercise saccadic control [48]. SPEMs might reflect the integration of cortical and cerebellar networks that support the prediction processes that underlie target tracking accuracy [57, 58]. Thus, the accuracy of eye movements at tracking reflects the functions of brain networks that enable complex goal-directed behavior.

Attention disruption is associated with dysfunction of various modulating mediator systems. Taking this fact into consideration, Siever et al. [59] studied the effect of amphetamine (a psychotropic substance that can affect the attention) on the accuracy of SPEMs in patients with OCD and with bipolar affective disorder. It was shown that amphetamine did not significantly affect the accuracy of SPEMs in these groups, however, patients with OCD demonstrated an effect of improving attention in the form of a significant negative dependency between the initial impairment of SPEMs and changes in target tracking accuracy. Consequently, based on the mentioned study, one cannot claim that patients with OCD have endophenotypic disorders in the system of SPEMs, and, thus, a deficit of involuntary attention [59].

Clementz et al. [60] found that moderate movement deficiency in patients with OCD occurred only during pursuit of fast-moving targets (24°/s). At a lower target movement speed (9 and 12°/s), no differences with the control group were identified [60].

Pallanti et al. [52] found a significant decrease in target tracking accuracy, as well as an increase in frequency and amplitude of anticipatory saccades during tracking task in patients with OCD compared with the control group. Lencer et al. [53] found a lower average speed in the foveafugal tracking task in both patients with OCD and those with schizophrenia and affective disorders. However, the post-saccadic eye speed in this task decreased only in patients with schizophrenia and affective disorder. In the foveapetal task, the average tracking speed was reduced only in patients with schizophrenia. The observed deficiencies in patients with OCD, schizophrenia, and affective disorders are indicative of the frontal lobes dysfunction, including the frontal eye fields. Unlike in OCD, in schizophrenia and affective disorders, a decrease in post-saccadic tracking initiation might indicate a disruption in the interaction between the smooth pursuit system and the saccadic system [53]. Farber et al. [55] in their study, on the contrary, demonstrated that patients with OCD and the control group had statistically similar tracking response times and average tracking acceleration during open and closed circuit tasks. Similarly, Bey et al. [61] showed that patients with OCD and their healthy relatives did not differ in performance in standard or predictive SPEMs.

Damilou et al. [62] identified high frequency of intrusive saccades during smooth pursuit tasks in patients with OCD and schizophrenia compared to healthy control group representatives. At that, unlike patients with schizophrenia, patients with OCD did not show an expressed decrease in acceleration during tracking. Therefore, these disorders have both common and specific impairments of the neurobiological mechanisms that control eye movements.

All the mentioned research studied the same type of eye movements. However, according to the available data, it is difficult to determine the nature of the deficiency of SPEMs in patients with OCD due to methodological differences, different research goals, studied parameters, as well as a small number of participants in the experimental sample.

### Studies of anti-saccades

Eye movement tasks can be used as model techniques for studying cognitive control. In psychopathology (including OCD), cognitive control is vulnerable to any voluntary action. Ability to voluntarily suppress dominant or reflex responses to perform a planned response being voluntary inhibition of the primary response is fundamental to the function of cognitive control [63]. One can assume that impaired performance in cognitive control tasks confirms impaired activity of pathways in the cortico-striatal-thalamic-cortical circuit in patients with OCD. Experimental paradigm with anti-saccades is one of the well-known techniques to study functions of cognitive control using the eye movement data.

In an anti-saccade task, the test participants must suppress the reflex eye movement (prosaccade) to a visual stimulus and make a voluntary movement to the opposite point in the visual space. An erroneous reaction is the inability to suppress the reflex reaction of eye movements to a peripheral stimulus. These errors are usually followed by a corrective saccade, indicating that the instruction was understood, but the reflex response failed to be suppressed [19].

Authors studying eye movements using the anti-saccade paradigm usually compare characteristics of anti-saccades and visually guided saccades to determine the neurophysiological mechanisms and brain structures that are involved in the processes of movement inhibition and their differences from the processes participating in the generation of saccades to visual stimuli. Anti-saccades have a longer latency than visually guided saccades to stimuli. Successful execution of anti-saccades requires two processes: suppression of the reflex saccade to the point of the peripheral stimulus and voluntary eye movement to a point mirrored to the peripheral stimulus in the visual space. Typical indicators in the anti-saccade task are errors, namely saccades in the wrong direction (reflecting problems with inhibitory control: inability to suppress an inappropriate visual response is most likely due to a lack of top-down control) and saccade latency [64]. In general, violations in the suppression of an irrelevant response are associated with dysfunctions of the frontal eye fields.

While performing visually guided saccades patients with OCD do not demonstrate differences compared to healthy individuals in terms of the number of errors and the extent of saccade latency [46], studies using anti-saccade tasks show a greater variability in results.

For instance, Maruff et al. [65] did not identify any differences in the amount of anti-saccadic errors in patients with OCD and healthy controls. The extent of the latency of visually guided saccades in the groups also did not differ, however, the extent of the latency of anti-saccades and voluntary saccades was significantly greater in patients with OCD than in the control group. The authors noted that in the absence of a goal an inhibitory deficiency was not confirmed, as patients with OCD encountered difficulties only in performing those tasks that required them to control movements based on their internal idea of the task goal. In tasks where the target remained visible throughout the test, no difference was found in the latency of saccades in patients with OCD and healthy individuals. Based on these data, Maruff et al. [65] suggested that cognitive deficiencies in OCD might be associated with difficulty to control behavior in the absence of an internal understanding of the task goal. This correlates with behavioral patterns in OCD — doubts and the fear of uncertainty.

An increase in anti-saccade latency in patients with OCD when performing anti-saccade and voluntary saccade tasks was found by van der Wee et al. [66]. It was suggested that patients with OCD do not have severe impairment of eye movement inhibition but might have an impaired ability to initiate a voluntary saccade to an imaginary target. In another study [67], no change in latency in patients with OCD was found; at that, an increased error rate was observed when performing tasks on anti-saccades compared with the healthy control group. A recent study by Narayanaswamy et al. [68] revealed a high percentage of errors and a less accurate end position of the eyes in an anti-saccade task in patients with OCD compared with the healthy control group; however, the authors did not find a significant correlation between anti-saccade parameters and disorder severity. The greater number of errors and the longer latency in patients with OCD in the mentioned studies indicate the impairments in functions of inhibitory control typical for this disorder.

In their study, Lennertz et al. [69] demonstrated that patients with OCD and their healthy first-degree relatives had an increased error rate in the anti-saccade task, as well as an increased anti-saccade latency compared to the control group of healthy individuals. This study provided the first evidence in favor of a candidate OCD endophenotype. Kloft et al. [47] later confirmed that healthy first-degree relatives of patients with OCD have a longer latency of voluntary saccades compared to healthy individuals. The same authors in their earlier study [70] showed that there were no differences in the error rate and in the latency when performing an anti-saccade task in patients with OCD and in healthy controls.

In a study of children diagnosed with OCD, Rosenberg et al. [71] found that such patients had more suppression failures than the control group, but only when peripheral visual targets were located close to the central fixation point; in case of the delayed response task, no significant differences between the groups were identified. The authors believe that the underlying impairment of inhibitory control in OCD might be the basis for the repetitive behavior typical for this disorder and associated with abnormalities in the orbitofrontal-ventral-striatal circuits. Moreover, they found that the response suppression deficiency was due to severity of symptoms in children with OCD. However, Ray et al. [72] in their study found that children diagnosed with OCD and the control group of healthy children showed no significant difference in terms of error rate, peak speed, shift of fixations at the control point, change in latency in tasks for prosaccades and anti-saccades. Thus, the results of this study do not confirm that eye movement impairments can be used as diagnostic biomarkers in children with OCD. However, children data cannot fully correlate with data of adult patients with OCD, as many measures of eye movement change as the brain develops and grows. In our opinion, it is necessary to study the characteristics of prosaccades and anti-saccades in various age groups.

Comparison of characteristics of anti-saccades in OCD and other mental illnesses appears to be of interest. For instance, McDowell et al. [73] in their study of patients with schizophrenia using an anti-saccadic task analyzed whether the error rate and latency were specific to this disease. The study results showed that the length of the saccade latency in patients with schizophrenia differed significantly when compared with patients with OCD and the healthy control group. In addition, patients with schizophrenia showed fewer corrective saccades than patients with OCD and healthy individuals. Spengler et al. [74] found that in an anti-saccade task the error rate and the length of the saccade latency demonstrated by patients with OCD did not differ from those in the healthy controls, in contrast to patients with schizophrenia. Damilou et al. [62] identified common and specific patterns of eye movement disorders in patients with schizophrenia and patients with OCD. The overall pattern of performance deficiency in the mentioned groups of patients included a higher error rate in the anti-saccade task, increased latency of corrective anti-saccades compared with the healthy control group. This common pattern might be related to impaired cognitive control that is seen in both diseases. At the same time, only patients with schizophrenia showed a specific increase in the latency of corrective anti-saccades and intrasubject variability in the latency in case of errors in prosaccades. A specific deficiency in fixation stability (an increase in the frequency of intrusive saccades along with active fixation) was observed only in patients with OCD, which the authors believed to indicate a deficiency in the frontal-striatal pathway that controls eye fixation.

During a study of eye movements using the anti-saccade task, it was established that such indicators as the length of the latency of anti-saccades and the error rate are higher in patients with OCD compared to patients with generalized anxiety disorder (GAD) [75], despite the fact that earlier (according to ICD-10 and DSM-4) these disorders were in the same group of diseases. At the same time, it was found that the voluntary saccade latency in patients with OCD is shorter compared to healthy individuals, and also it is significantly shorter than their own anti-saccade latency. These results serve as a possible justification for habitual compulsive manifestations and behavioral rigidity in patients with OCD, in contrast to patients with GAD, where there are no behavioral reactions in the form of obsessions [75]. Although GAD might genetically overlap with OCD [76], a recent study [77] demonstrated that cognitive control ability improves under stress in patients with GAD in contrast to patients with OCD.

Inconsistent research results (see Appendix 2) are probably due to studying small samples. However, Bey et al. [24] confirmed the data of many earlier studies on a sufficiently large sample (169 patients with OCD) and demonstrated the following moderate impairments in anti-saccade control in patients with OCD: increased anti-saccade latency, intrasubject variability of anti-saccade latency, and an increase in anti-saccade errors compared with the healthy control group. The latter effect was a result of errors in express saccades, though patients with OCD did not significantly differ from the control group in terms of errors in regular saccades. These parameters in the form of errors during express and regular saccades in patients with OCD had not been studied before, although they can be used to identify certain neural mechanisms of inhibitory control which are impaired in OCD. Healthy relatives of patients with OCD also showed an increased error rate and increased latency’s variability when performing an anti-saccadic task, which might indicate an assumed OCD endophenotype.

Most studies of anti-saccades (see Appendix 2) specify impairments in inhibitory control in patients with OCD, manifested by an increase in latency and error rate, which might correlate with an imbalance in transmission of excitation and inhibition in the cortico-striatal-thalamic-cortical circuit.

### Eye movement paradigms aimed at studying working memory and attention

WM and attention are part of the human cognitive control functions. WM refers to the cognitive ability to store a limited amount of information for a short period of time and manipulate it momentarily in relation to immediate stimuli to achieve a required goal that is inaccessible for immediate perception. As WM supports volitional task-oriented actions, WM study will probably contribute to understanding of obsessions in OCD, especially taking into account that some studies indicate that rituals (compulsive actions) might be a consequence of WM deficiency [46].

One of the standard experimental schemes aimed at studying memory is the memory-guided saccade paradigm. A person is asked to look at the central fixation point, during the presentation of which a target appears in the peripheral visual field. The person must remember the location of the target, while not looking at it (without a saccade to it). When the fixation point disappears, the person is to perform a saccade to the memorized location. The time between the disappearance of the target and the moment when the subject needs to perform the saccade (delay period) might vary depending on the different load on the visuospatial WM. Typical indicators studied in the memory-guided saccade task include accuracy, the number of anticipatory saccades, and the latency’s length [56].

When performing the memory-guided saccade task, the dorsolateral prefrontal cortex, the anterior cingulate cortex, and the supplementary eye field are activated [78]. Based on these data, it can be assumed that the deficiency in performance of the memory-guided saccade task is associated with an impaired executive function of the frontal cortex.

Some studies refer to the memory-guided saccade paradigm as an oculomotor delayed response task. It is not often used when studying eye movement reactions in patients with OCD, in contrast to the anti-saccade task. It was established that the number of errors in the fixation task is slightly higher in patients with OCD compared to the control group, with errors seen only when the peripheral stimulus was close to the fixation point. The overall accuracy of saccades with respect to the memorized target locations did not differ between patients with OCD and the control group, although there was a trend toward increased latency in OCD [71]. There is a need to conduct additional studies with the memory-guided saccade task to draw definitive conclusions about WM impairment in patients with OCD, as it has been established for other paradigms that visuospatial memory impairment in patients with OCD is one of the most frequently reported cognitive deficiencies [79-81].

Jaafari et al. [82] studied verbal, visuospatial components of WM in patients with OCD using the reading span test, backward location span test, and image comparison test. The WM volume in these tasks was reduced in patients with OCD, they performed more saccades for image comparison than the control group. Besides, the indicators of the number of saccades in patients with OCD were increased when comparing different rather than identical images [82, 83]. This contradicts the data of Rotge et al. [83], who demonstrated that patients with OCD performed more testing saccades when the images were identical. These differences might be a result of different ways of presenting two images (sequential or simultaneous) and different assessments of the testing behavior (number of voluntary checks compared to number of eye movements between images). In addition, there was a strong correlation between participants’ WM volume and performance: low WM volume was associated with longer time when comparing different versions of images and with more eye movements between two images, i.e. with more intensive verification [82, 83]. In general, the mentioned studies might indicate that the intensity of patients’ behavioral symptoms and, in particular, verifiability is closely related to WM impairment.

Lee et al. [84] also demonstrated that patients with OCD more frequently return to the words they read than individuals in the control group and gaming addicts. This might be due either to inhibited information processing or to a WM deficiency. Although incorrect results could also be due to severity and manifestation of OCD symptoms, as well as use of medicinal products.

Harkin et al. [85] in their study established that individuals with a high level of rechecking subtype (checkers) more frequently and longer remain fixed on misleading information than the control group. In particular, checkers spend more time checking stimuli locations, including those which were actually empty at presentation. Apparently, failure of attention and WM during misleading tasks results in checkers over-checking their percepts in WM, comparing them even with empty, non-informative locations [86, 87].

It has been hypothesized that WM impairment in OCD might be due to difficulties in focusing on relevant information and inability to suppress irrelevant stimuli. This, in turn, can overload the WM with input data that are insignificant for the current task and result in the WM deficiency [82, 88]. In this case, according to the Harkin and Kessler model, WM impairment in patients with OCD is secondary to executive dysfunction [89].

Several studies emphasized that patients with OCD have difficulties with ignoring irrelevant stimuli and show impaired selective attention abilities. The reviews by Kashyap et al. [90] and Snyder et al. [91] demonstrated that the attention deficiency in patients with OCD is mainly a by-product of their impaired cognitive control functions, and it indicates a decrease in skills related to organization of the input data and optimization of cognitive resources. The latter assumption is consistent with the conclusion of the review on neuroimaging studies of patients with OCD stating the impaired sensory gating in OCD [13].

Several studies based on the visual search paradigm examined only the response time and accuracy with no analysis of patients’ eye movements. The visual search paradigm provides for investigation of attention through eye movements and consists in searching for a target object among other visual stimuli, called “distractions” [92, 93]. For instance, Botta et al. [94] showed that patients with OCD took longer time to perform visual word searches and more often fixed their eyes on non-target words compared to healthy subjects. Different types of distraction words have different effects on visual search in patients with OCD and the control group. This suggests that attention control mechanisms differ between patients with OCD and healthy individuals. Healthy controls fixed more on words visually similar to the target stimulus [95], while patients with OCD — on all distracting (neutral, spelling, and semantic) factors, at that patients with OCD less frequently fix their eyes on distractions related to intrusive ideas [94]. These data suggest problems in optimization of attention resources allocation in patients with OCD, as they are unable to adequately suppress stimuli which are not related to the task. Switching attention to any distracting stimulus might explain why patients with OCD are generally slower and less efficient than healthy individuals in visual search tasks. It is of interest that patients with OCD made fewer fixations on words related to intrusive ideas [94]. This might be due to the fact that, firstly, according to Greene et al. [96], during visual search, a person fixes less on familiar distractions than on unfamiliar ones. And secondly, patients with OCD might unconsciously avoid information related to their obsessions. It is interesting to note that Najmi et al. [97] in the study in patients with OCD showed that the speed of eye approach to images associated with pollution was lower than in the control group, and at the same time, patients with OCD did not avoid these images with their eyes.

Data on specific aspects of WM in patients with OCD obtained through neuropsychological tests are contradictory and ambiguous [98, 99]. However, most studies of WM based on eye movements confirm that WM volume is lower in patients with OCD. In turn, this might be one of the causes of compulsive behavior (rituals). However, it is not clear whether the WM impairment is the primary factor triggering compulsive behavior, or it is secondary to executive functions, including selective attention. Difficulties in focusing attention and suppression of irrelevant stimuli that can be traced in patients with OCD, in our opinion, reflect an overall dysfunction of cognitive control. It should also be noted that the results of the mentioned studies also depend on the extent to which the stimuli provided are significant for the test subject personally. OCD is often accompanied by anxiety, and the state of anxiety of patients with OCD during the experiment can lower their WM and impair the overall performance in specific tasks. There is a need to conduct further studies preceded by analysis of situational and personal anxiety in order to determine the unambiguity and validity of the data on impaired cognitive control functions, including WM, in patients with OCD.

## Conclusion

Oculography and eye tracking techniques are actively used to assess preservation of cognitive control in different clinical groups, including in patients with obsessive-compulsive disorder. While studying eye movement functions in patients with this disorder, the following paradigms are most often used: SPEM tasks, memory-guided saccades, and anti-saccades. Data on SPEM tasks are currently contradictory. Studies of memory-guided saccades have not shown a reliable impairment of working memory in patients with obsessive-compulsive disorder, although the relation of oculomotor parameters with working memory and selective attention impairments in this pathology was demonstrated. However, study results might be influenced by premorbid personal traits associated with anxiety, which should be taken into account in the future. In many studies based on anti-saccadic task in patients with obsessive-compulsive disorder, impairments in the inhibitory control function were identified. Similar disorders, manifested in increased latency and a higher error rate in anti-saccades, were found in the said patients’ first-degree relatives, which might be a potential endophenotype of obsessive-compulsive disorder. Future confirmation of these results in experiments with complex anti-saccadic tasks using images of various modalities (taking into account the increased anxiety in patients with obsessive-compulsive disorder as the basis of the disorder) might contribute to validation of specific markers of obsessive-compulsive disorder.

## Appendix 1

**Table:** Summarized information on studies selected to review the specific aspects of eye movement reactions in patients with obsessive-compulsive disorder (OCD)

No.	References	Number and characteristics of the test subjects	Recorded eye movement activity (task)	Function	Conclusion
1	Siever et al., 1987 [59]	8 — OCD 5 — bipolar affective disorder	Smooth pursuit eye movements	Involuntary attention influenced by amphetamine	Amphetamine does not significantly affect the accuracy of smooth pursuit eye movements in OCD and bipolar affective disorder. However, in patients with OCD, there is an effect of attention improvement in the course of amphetamine treatment, consisting in a significant negative dependency between the initial impairment of smooth pursuit eye movements and changes in target tracking accuracy
2	Nickoloff et al., 1991 [54]	8 — OCD 12 — control group	Smooth pursuit eye movements	Involuntary attention	Patients with OCD and control group did not differ in performance while fulfilling the task of smooth pursuit eye movements
3	Sweeney et al., 1992 [50]	17 — OCD 25 — control group	Smooth pursuit eye movements	Involuntary attention	Patients with OCD showed less accuracy in the task related to tracking and an increased frequency of “rectangular” saccades; there is also an increase in anticipatory saccades not observed
4	Gambini et al., 1993 [51]	23 — OCD 27 — control group	Smooth pursuit eye movements	Involuntary attention	Patients with OCD demonstrated less accuracy in the task related to tracking compared to test individuals from the control group
5	Pallanti et al., 1996 [52]	21 — OCD 21 — control group	Smooth pursuit eye movements	Involuntary attention	Patients with OCD demonstrated a significant reduction in tracking accuracy, as well as an increase in the frequency and range of anticipatory saccades, in contrast to the control group
6	Clementz et al., 1996 [60]	12 — OCD 12 — control group	Smooth pursuit eye movements	Involuntary attention	When tracking fast moving targets (24°/s), patients with OCD demonstrated a moderate deficiency, whereas in case of a slower target speed (9 and 12°/s), no differences with the control group were recorded
7	Farber et al., 1997 [55]	20 — OCD 20 — schizophrenia 20 — control group	Smooth pursuit eye movements (open and closed cycle tasks)	Involuntary attention	Patients with OCD and control group demonstrated statistically similar pursuit reaction time and average eye acceleration during open and closed cycle tasks, in contrast to patients with schizophrenia
8	Lencer et al., 2004 [53]	18 — OCD 16 — schizophrenia 15 — affective disorder 23 — control group	Smooth pursuit eye movements (foveafugal and foveapetal tasks)	Involuntary attention	Patients with OCD and people with schizophrenia and affective disorders demonstrated lower steady-state velocity in the foveafugal step-ramp task. The post-saccadic eye velocity in the foveafugal task decreased only in patients with schizophrenia and affective disorder. In the foveapetal step-ramp task, the average steady-state velocity was decreased only in patients with schizophrenia
9	Damilou et al., 2016 [62]	34 — OCD 44 — schizophrenia 45 — control group	Smooth pursuit eye movements (standard and prospective tracking), anti-saccades	Involuntary attention	Patients with OCD demonstrated a high frequency of intrusive saccades compared to the control group. Here, in contrast to patients with schizophrenia, patients with OCD did not show an apparent decrease in acceleration during tracking. The overall performance deficiency in schizophrenia and OCD included a higher errors rate in the anti-saccade task, increased latency of corrective anti-saccades compared to the control group
10	Bey et al., 2019 [61]	168 — OCD 93 — relatives of patients with OCD 171 — control group	Smooth pursuit eye movements	Involuntary attention	Patients with OCD and their healthy relatives did not differ in performance in standard or predictive smooth pursuit eye movements
11	Jaafari et al., 2011 [23]	—	Anti-saccades, visually induced saccades	Voluntary inhibitory control	Patients with OCD demonstrated no differences compared to healthy test individuals when performing visually induced saccades in terms of the number of errors and the extent of saccades latency, at that studies involving anti-saccade tasks demonstrate a greater variability of results
12	Tien et al., 1992 [49]	11 — OCD 14 — control group	Anti-saccades, visually-guided saccades	Involuntary attention, voluntary inhibitory control	Patients with OCD demonstrated a higher rate of errors and inaccurate saccades in the anti-saccade task compared with the control group; here, the groups did not differ in reaction time, saccade velocity, accuracy when performing tasks related to visually-guided saccades
13	McDowell et al., 1997 [73]	12 — OCD 12 — schizophrenia 12 — control group	Anti-saccades	Voluntary inhibitory control	In patients with schizophrenia, the value of the latency of anti-saccades was significantly different compared with patients with OCD and the healthy control group. Patients with schizophrenia showed fewer corrective saccades than patients with OCD and the control group
14	Rosenberg et al., 1997 [71]	18 — OCD 18 — control group	Anti-saccades, memory-guided saccades	Voluntary inhibitory control in children	Patients with OCD showed more response suppression failures than the control group when peripheral visual targets were located close to the central fixation point, whereas in case of the delayed response task there was no significant difference seen. Memory-guided saccades: in patients with OCD, the number of errors in the fixation task was insignificantly higher comparing to the control group, but errors occurred only when the peripheral stimulus was close to the fixation point. The overall accuracy of saccades with respect to the memorized target locations did not differ between patients with OCD and the control group, although patients with OCD demonstrated a trend to an increased latency
15	Maruff et al., 1999 [65]	12 — OCD 12 — control group	Anti-saccades, visually-guided saccades, voluntary saccades	Voluntary inhibitory control	There were no differences in rates of anti-saccadic errors for patients with OCD and the controls group. The latency of visually-guided saccades also did not differ between the groups; however, the latency of anti-saccades and voluntary saccades were significantly longer in patients with OCD
16	Spengler et al., 2006 [74]	22 — OCD 21 — schizophrenia 24 — control group	Anti-saccades	Voluntary inhibitory control, suppression function	The error rate and latency of anti-saccades in patients with OCD did not differ from the control group, in contrast to patients with schizophrenia
17	van der Wee et al., 2006 [66]	14 — OCD 14 — control group	Anti-saccades, voluntary saccades	Voluntary inhibitory control	Patients with OCD showed latency deceleration in anti-saccadic tasks, there were no differences found for other parameters
18	Kloft et al., 2011 [70]	30 — OCD 30 — control group	Anti-saccades	Voluntary inhibitory control	Such parameters as the error rate and latency in patients with OCD and the control group did not differ
19	Lennertz et al., 2012 [69]	30 — OCD 30 — relatives of patients with OCD 30 — control group	Anti-saccades	Voluntary inhibitory control	Patients with OCD and their relatives demonstrated an increased error rate and an increase in anti-saccade latency compared to the control group
20	Kloft et al., 2013 [47]	22 — OCD 22 — relatives of patients with OCD 22 — control group	Anti-saccades, voluntary saccades	Voluntary inhibitory control	Patients with OCD and their relatives demonstrated an increased error rate in anti-saccades tasks and an increase in the latency of anti-saccades compared with the control group. Relatives of patients with OCD showed a longer latency of voluntary saccades compared to the control group
21	Agam et al., 2014 [67]	21 — OCD 20 — control group	Anti-saccades	Voluntary inhibitory control	Patients with OCD demonstrated no change in latency. However, they had an increased error rate compared to the control group
22	Bey et al., 2018 [24]	169 — OCD 183 — control group 100 — relatives of patients with OCD	Anti-saccades	Voluntary inhibitory control	In case of OCD, moderate performance impairments in the anti-saccadic task were identified: increased latency, intra-subject variability in anti-saccade latency, and increased frequency of anti-saccadic errors compared with the control group. The effect of the increased frequency of anti-saccadic errors was caused by errors associated with express saccades, while the patients’ results did not differ much from the control group in terms of errors in case of regular saccades. Healthy relatives of patients with OCD demonstrated an increased error rates, as well as an increased variability in anti-saccade latency
23	Ray et al., 2019 [72]	36 — OCD 31 — control group	Anti-saccades, voluntary saccades	Voluntary inhibitory control in children	There was no significant difference between children diagnosed with OCD and the control group in terms of error rate, peak rate, shift of fixation on the control point, latency changes in anti-saccade tasks and voluntary saccades
24	Hu et al., 2020 [75]	25 — OCD 25 — generalized anxiety disorder 25 — control group	Anti-saccades	Voluntary inhibitory control	Anti-saccade latency and error rates are higher in patients with OCD compared to patients with generalized anxiety disorder
25	Narayanaswamy et al., 2021 [68]	65 — OCD 57 — control group	Anti-saccades	Voluntary inhibitory control	Patients with OCD demonstrated high percentage of erroneous saccades and less accurate final eye position compared with the control group
26	Rotge et al., 2008 [83]	50 — OCD 50 — control group	“Image comparison” task	Working memory	Patients with OCD performed more testing saccades when the images were identical
27	Harkin et al., 2012 [85]	18 — people who are highly likely to double-check 17 — people who are unlikely to double-check, control group	“Memorizing location” task	Working memory	People who are highly likely to double-check (checkers) more often and longer fix on misleading information than the control group. In particular, checkers spend more time checking stimuli locations, including locations that were actually empty during presentation
28	Jaafari et al., 2013 [82]	32 — OCD 32 — control group	“Memorizing during reading”, “Memorizing reverse location”, “Images comparison” tasks	Working memory	Patients with OCD demonstrated a decreased capacity of working memory in the tasks, there were more saccades at images comparison than in the control group. Here, the indicators of the saccade number in patients with OCD were increased when comparing different, rather than identical images
29	Lee et al., 2018 [84]	20 — OCD 28 — gaming addiction 24 — control group	“Memorizing during reading” task	Working memory	Patients with OCD demonstrated significantly more returns to words during reading than the control group and gaming addicts
30	Botta et al., 2018 [94]	36 — OCD 36 — control group	“Visual search of words” task	Attention	Patients with OCD needed more time to perform visual searches of words than healthy participants; they were also more inclined to fix their eyes on non-target words

## Appendix 2

**Table:** Results of studies, where the anti-saccadic task is used, in patients with obsessive-compulsive disorder (OCD)

No.	References	Number of test subjects (OCD/control)	Results: latency	Results: errors frequency
1	Tien et al., 1992 [49]	11/14	—	OCD > control
2	McDowell et al., 1997 [73]	12/12	OCD > control	OCD = control
3	Rosenberg et al., 1997 [71]	18/18	OCD = control	OCD > control (8°)
				OCD = control (16°)
				OCD = control (24°)
4	Maruff et al., 1999 [65]	12/12	OCD > control	OCD = control
5	Spengler et al., 2006 [74]	22/24	OCD = control	OCD = control
6	van der Wee et al., 2006 [66]	14/14	OCD > control	OCD = control
7	Kloft et al., 2011 [70]	30/30	OCD = control	OCD = control
8	Lennertz et al., 2012 [69]	30/30	OCD > control (PS)	OCD > control
9	Agam et al., 2014 [67]	21/20	OCD = control (EEG)	OCD = control (EEG)
			OCD = control (fMRI)	OCD > control (fMRI)
10	Damilou et al., 2016 [62]	34/45	OCD = control	OCD > control
11	Bey et al., 2018 [24]	169/183	OCD > control	OCD > control
12	Ray et al., 2019 [72]	36/31	OCD = control (children)	OCD = control (children)
13	Hu et al., 2020 [75]	25/25	OCD > control	OCD > control
14	Narayanaswamy et al., 2021 [68]	65/57	—	OCD > control
